# A novel plate design for anatomic reduction of posterior acetabular column and wall fractures: A retrospective comparative study of the Paseos plate versus the pelvic reconstruction plate

**DOI:** 10.1097/MD.0000000000050011

**Published:** 2026-07-31

**Authors:** Emre Gültaç, Fatih İlker Can, Fatih Özyer, Mehmet Arazi, Osman Kurtuluş

**Affiliations:** a Department of Orthopedics and Traumatology, Faculty of Medicine, Mugla Sitki Kocman University, Muğla, Turkey; b Department of Orthopedics and Traumatology, Fethiye State Hospital, Muğla, Turkey; c Department of Orthopedics and Traumatology, Private Farabi Hospital, Konya, Turkey; d Private Clinic, Orthopedics and Traumatology, Muğla, Turkey.

**Keywords:** novel acetabular plate design, plate and screw fixation, posterior acetabular fractures, surgical outcomes

## Abstract

This study endeavors to compare the clinical and radiological outcomes of 2 different types of plate fixation for posterior acetabular wall and/or column fractures, transverse and posterior wall fractures. We designed an anatomical plate that is easy to apply for acetabular posterior wall and column fractures and can simultaneously fix the column and wall as support with the buttressing effect. Weight-bearing and bending biomechanical tests were performed for the development of the plate. The 39 acetabular fractures operated between January 2013 and June 2017 were divided into 2 groups. Group 1 (n = 17) received treatment using the Paseos anatomical plate, while Group 2 (n = 22) with conventional reconstruction plates. Merle d’Aubigné and Postel score and Matta reduction criteria were utilized for the functional and radiological evaluations. According to Matta reduction criteria, in Group 1, anatomical reduction was achieved in 14 patients (82.3%), good reduction in 2 patients (11.7%), and poor reduction in 1 patient (5.9%). In Group 2, anatomical reduction was observed in 19 patients (86.4%), good reduction in 2 patients (9.1%), and poor reduction in 1 patient (4.5%). At the 12-month follow-up, functional outcomes were evaluated using Merle d’Aubigné and Postel scoring system. In Group 1, excellent scores were recorded in 13 patients (76.4%), good scores in 3 patients (17.6%), and fair scores in 1 patient (5.9%). In Group 2, excellent scores were observed in 19 patients (86.4%), good scores in 2 patients (9.1%), and fair scores in 1 patient (4.5%). The median operation duration in Group 1 was significantly lower than Group 2 (*P* < .001). Paseos plate demonstrated clinical and radiological outcomes comparable to conventional reconstruction plates. Due to its special anatomical structure, it can support a wide bony area with its buttress effect and also it can be applied directly without the need for reshaping during surgery, thus shortening the surgical duration.

## 1. Introduction

Acetabular fractures are complex injuries commonly associated with high-energy trauma and remain challenging because of the need for accurate reduction and stable fixation. The involvement of the posterior column and wall occurs in approximately 35% of these fractures.^[1]^ Surgical intervention is warranted for all fractures with a displacement exceeding 2 mm on the acetabular joint surface.^[2]^ Historically, the primary objective in treating such fractures was solely to preserve patient life, given the massive bleeding at fracture sites and associated organ injuries. However, contemporary consensus emphasizes that the anatomical reduction and stabilization of pelvic ring fractures constitute the pivotal step in mitigating life-threatening hemorrhage. Recent advancements, including 3-dimensional (3D) radiological imaging techniques, have significantly enhanced our understanding of fracture location and morphology, enabling more precise classifications. Novel surgical implants and techniques have contributed to improved survival and, in many cases, better functional recovery.

Posterior acetabular wall and column fractures remain technically demanding because of the complex 3D anatomy of the acetabulum and the multiplanar extension of fracture fragments. In conventional fixation methods, reconstruction plates often require extensive intraoperative contouring to match the patient-specific pelvic anatomy, and in complex fracture configurations, multiple plates may be necessary to achieve sufficient buttress support and stability. This may result in increased operative time, technical difficulty, plate overlap, and challenges in obtaining stable fixation over a broad surface area. To address these limitations, we designed the Paseos plate as a pre-contoured anatomical fixation system specifically for posterior wall and column fractures. The plate was designed to provide a wider buttress surface, simultaneous support of the posterior wall and column, and the potential for stable single-plate fixation while reducing the need for repeated intraoperative bending and contouring. We hypothesized that these features could facilitate surgical application and potentially shorten operative duration while maintaining satisfactory clinical and radiological outcomes. Presently, pelvic reconstruction plates, characterized by varying configurations and angles, find widespread application, tailored to the specific fracture morphology and extent.^[3]^

Within the existing literature, numerous studies have elucidated the determinations and outcomes associated with pelvis reconstruction plates, a stalwart in the surgical management of acetabular posterior column and wall fractures.^[3–7]^ However, the intricate 3D anatomy of the pelvis, coupled with fracture morphologies that transcend a single plane, presents unique challenges. Notably, Lei et al delved into the distribution of stress and forces following the fixation of complex acetabular fractures, meticulously assessing their displacement response under load through finite element modeling. Their findings underscored the inadequacy of single-line fixations in complex fractures, emphasizing the superiority of expansive surface fixations and dual-column approaches.^[1]^

The aim of this study was to evaluate the clinical and radiological outcomes of a newly designed anatomical acetabular plate (Paseos plate) compared with conventional pelvic reconstruction plates in posterior acetabular wall and/or column fractures. We additionally aimed to assess whether the pre-contoured anatomical design could reduce operative duration. To our knowledge, there are limited clinical data evaluating anatomical single-plate fixation systems specifically designed for simultaneous posterior wall and column buttressing. After the necessary biomechanical strength studies, we utilized our novel plate design for fixating the posterior wall and column fractures and examined the clinical and radiological results afterward.

## 2. Methods

A retrospective analysis was conducted on 39 acetabulum fractures treated at our clinic between January 2013 and June 2017. Because of the retrospective design and the use of anonymized clinical data, formal institutional review board approval was not required according to institutional policy, and separate informed consent for the retrospective use of the data was waived; institutional permission for retrospective data use was obtained from the hospital administration. At the time of surgery, all patients had provided written informed consent for the operative procedure, which, in accordance with our routine surgical practice, also included being informed that a newly designed implant (the Paseos plate) would be used. The study was conducted in accordance with the Declaration of Helsinki. During the study period (January 2013–June 2017), a total of 66 patients with surgically treated acetabular fractures were retrospectively screened for eligibility. Of these, 9 were excluded because of isolated anterior column fractures, 6 because of concomitant ipsilateral lower-extremity fractures, 1 because of ipsilateral neurological injuries, and 11 because of incomplete medical records. After application of these exclusion criteria, 39 patients with complete 12-month clinical and radiological follow-up were included in the final analysis. Power analysis was not performed because of the retrospective design; the sample size was determined by the number of eligible patients treated during the study period. The cohort was then divided into 2 groups: Group 1 (17 patients) treated with the Paseos plate and Group 2 (22 patients) treated with conventional pelvic reconstruction plates. Patients were not randomized between implant groups. All surgical procedures were performed by the same surgical team throughout the study period. Implant selection was based on surgeon preference, implant availability, and the period during which the Paseos plate became available for clinical use. Conventional reconstruction plates were used routinely before the introduction of the Paseos plate, whereas eligible posterior wall and/or posterior column fractures treated after its introduction were managed with the Paseos plate according to the operating surgeon’s decision. All cases included in this study were attributed to traffic accidents or falls from significant heights. As part of our preoperative protocol, 3D computed tomography (CT) scans were routinely employed for all patients in conjunction with anteroposterior and 45° oblique Judet (obturator and iliac oblique) pelvic radiographs to classify the injuries according to the Judet–Letournel classification.^[8]^ The reduction quality was assessed using Matta reduction criteria (MRC)^[9]^ and the functional outcomes were examined using the Merle d’Aubigné and Postel scores (MDPS).^[10]^ These assessments were evaluated by a single orthopedic surgeon. Because the implant type was identifiable on postoperative radiographs and CT, blinding of the assessor to plate type was not feasible; this is acknowledged as a limitation of the study. The data used in the present study were retrospectively obtained from these standardized clinical and radiological follow-up records. Complications were evaluated during routine postoperative outpatient follow-up visits based on clinical examination and radiographic assessment. Infection was defined according to the presence of clinical signs requiring antibiotic treatment or surgical intervention. Heterotopic ossification was identified on follow-up radiographs, and screw loosening was determined radiographically by evidence of screw back-out, implant migration, or loss of fixation. Complication data were retrospectively obtained from standardized clinical follow-up records and radiographic evaluations.

### 2.1. Surgical approach

All patients underwent procedures under general anesthesia, receiving prophylactic first-generation cephalosporin (1 g administered approximately 1 hour before incision) preoperatively, alongside subcutaneous enoxaparin (Clexane, Aventis Intercontinental). The operative setting involved a lateral decubitus position on a radiolucent table. Employing the Kocher–Langenbeck approach uniformly across all fractures within this study, we meticulously exposed the fracture site, achieving reduction through a combination of direct and indirect manipulation. Subsequent fixation involved the routine utilization of lag screws to establish initial stability, facilitating the placement of the appropriately sized plate for definitive fixation.

### 2.2. Plate design

Historically, the fixation of acetabular fractures relied upon 3.5 mm screws and reconstruction plates, tailored to the anatomical intricacies of each fracture. However, when confronted with fracture fragments of varying sizes extending in divergent directions, the need for multiple plates arose, sometimes resulting in plate overlap and the inability to place multiple plates. Recognizing this challenge, we envisioned a more stable design: interconnecting these plates akin to branching structures, accommodating the fracture’s extension. Drawing from our surgical experience, we sought to achieve stability through a single newly designed anatomical plate intended to envelop the fracture fragments and provide adequate fixation.

**Figure 1.**
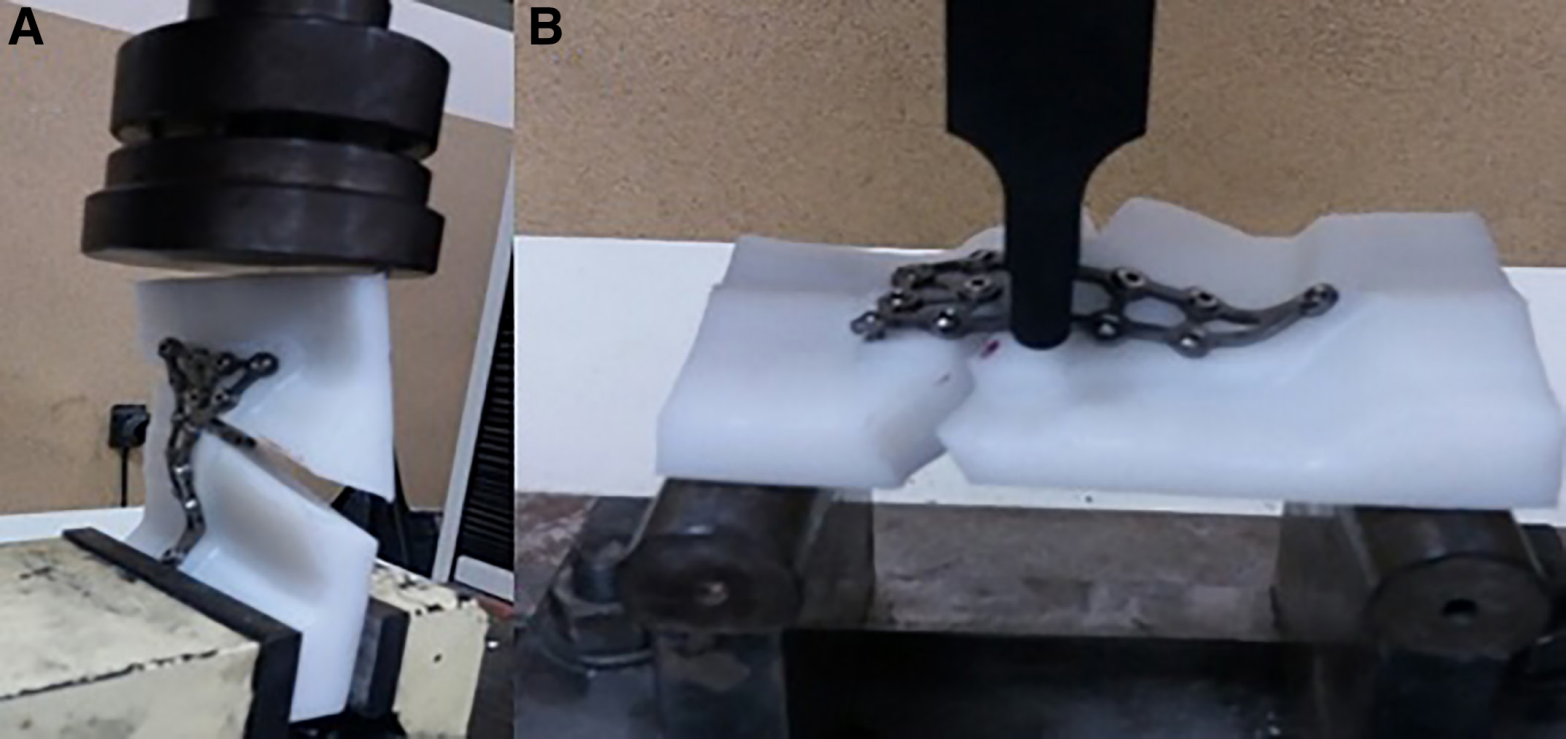
(A) The weight-bearing and (B) bending biomechanical tests of the Paseos plate.

**Figure 2.**
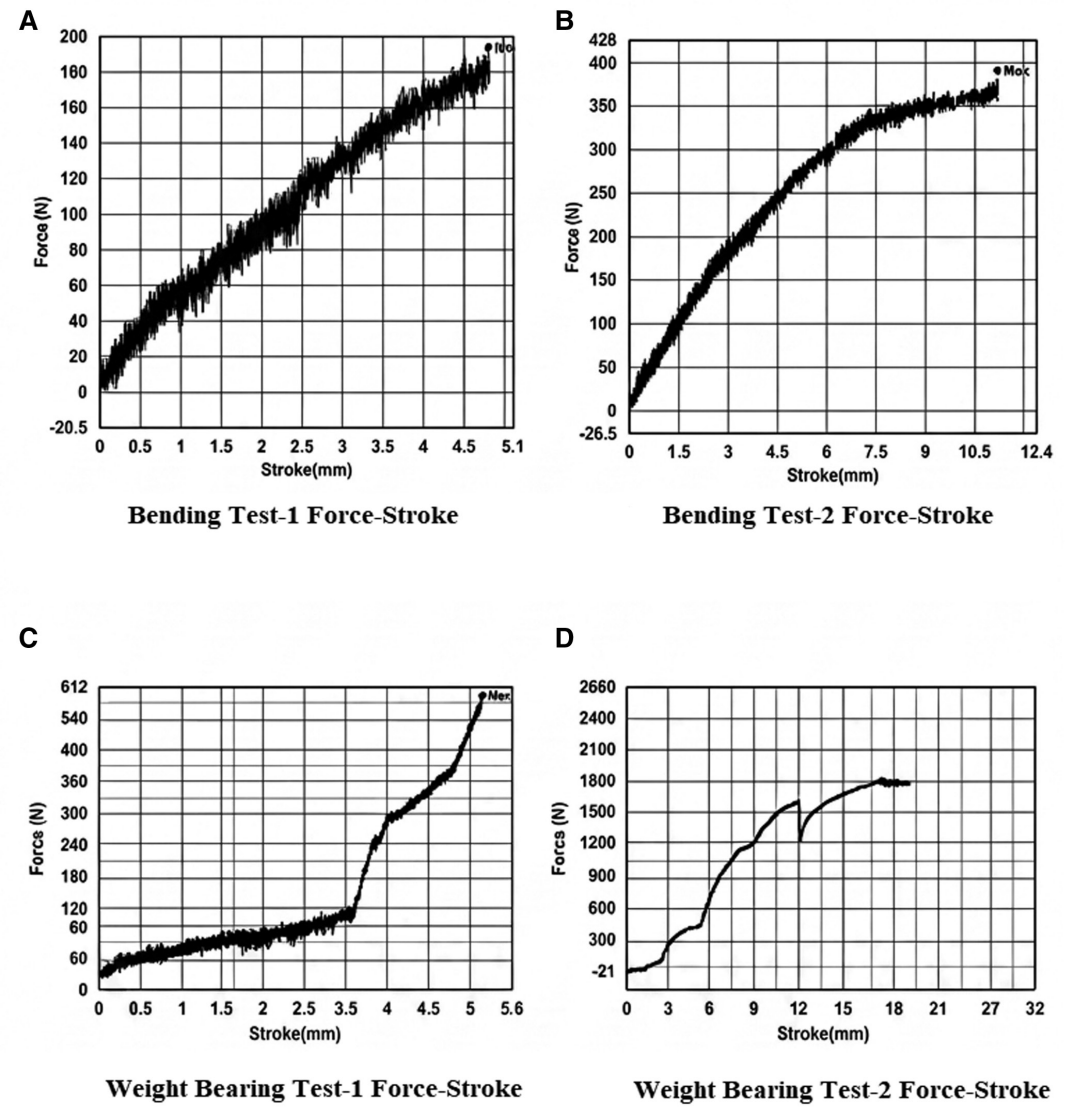
Force–stroke diagrams of the biomechanical tests of the Paseos plate. (A, B) Bending tests; (C, D) 2 separate weight-bearing (axial) tests. In each diagram, the x-axis represents stroke (displacement, mm) and the y-axis represents force (N); loading was applied at 1 mm/minute until the onset of failure. The maximum force sustained before failure was 390 N in the bending tests and 1800 N in the weight-bearing tests.

The Paseos plate design comprises 2 distinct arms extending upward. One arm parallels the acetabulum, while the other aligns with the greater sciatic notch. Anchored by a 2.7 mm long screw originating from the distal-inferior aspect of the acetabulum, the plate’s solid base curves to snugly fit the ischial tuberosity. Boasting 14 screw holes, it comprehensively envelops the posterior wall and column. Its curved form, reminiscent of the acetabular ring, even permits screw placement beyond the acetabulum into the ilium. To accommodate anatomical variations, we meticulously crafted 3 plate sizes. Following the design phase on anatomical models, we subjected the Paseos plate to preliminary biomechanical durability tests. Bending and compression loading assessments were conducted on fracture samples within the pelvis, under the auspices of Ege University Faculty of Engineering’s Mechanical Engineering Department. In these preliminary tests, the plate maintained its structural integrity within the tested loading range under both vertical and rotational loading (Figs. 1–2).

### 2.3. Biomechanical testing

The Paseos plate is a pre-contoured anatomical pelvic plate manufactured from implant-grade titanium alloy (Ti-6Al-4V; International Organization for Standardization 5832-3, Grade 5) by Prosim Uluslararasi Medikal Cihazlar İthalat İhracat Ltd. Şti. At the time of clinical use, the plate was used under institutional permission at the discretion of the operating surgeons; no separate device-specific regulatory (marketing) approval or prospective ethics committee approval was obtained for its clinical use. This is stated transparently and was in keeping with local institutional practice at the time. Biomechanical testing was performed on 4 Paseos plate samples manufactured from titanium alloy, mounted on synthetic bone (Sawbones) pelvic models, using a mechanical testing device under ambient laboratory conditions (room temperature). Two loading modes were applied: bending, with the construct positioned horizontally, and axial compression (weight-bearing), with the construct positioned vertically. The testing speed was standardized at 1 mm/minute, and force–stroke values were recorded continuously during loading. Failure was defined as the onset of macroscopic cracking (fracture initiation) of the construct, and the maximum force sustained before this point was recorded. Biomechanical testing was performed only for the Paseos plate construct during the developmental phase of the implant; conventional reconstruction plate constructs were not included. Therefore, these findings should be interpreted as preliminary developmental feasibility data rather than direct comparative biomechanical evidence.

### 2.4. Statistical analysis

Statistical analysis was performed using IBM SPSS version 22.0 software (IBM Corp.). Descriptive data were presented as mean ± standard deviation, median (min–max), or number and percentage, where appropriate. The normality of data distribution was evaluated using the Shapiro–Wilk test. As the data did not demonstrate a normal distribution, nonparametric statistical methods were employed for further analyses. Continuous variables between 2 independent groups were compared using the Mann–Whitney *U* test. The between-group difference in operative duration was estimated using the Hodges–Lehmann median difference with its 95% confidence interval, consistent with the Mann–Whitney *U* test, and the effect size was expressed as the rank-biserial correlation coefficient. Categorical variables, including MRC and Merle d’Aubigné–Postel outcome categories, were compared using the chi-square test or Fisher exact test, as appropriate. A *P* value of < .05 was considered statistically significant.

## 3. Results

**Figure 3.**
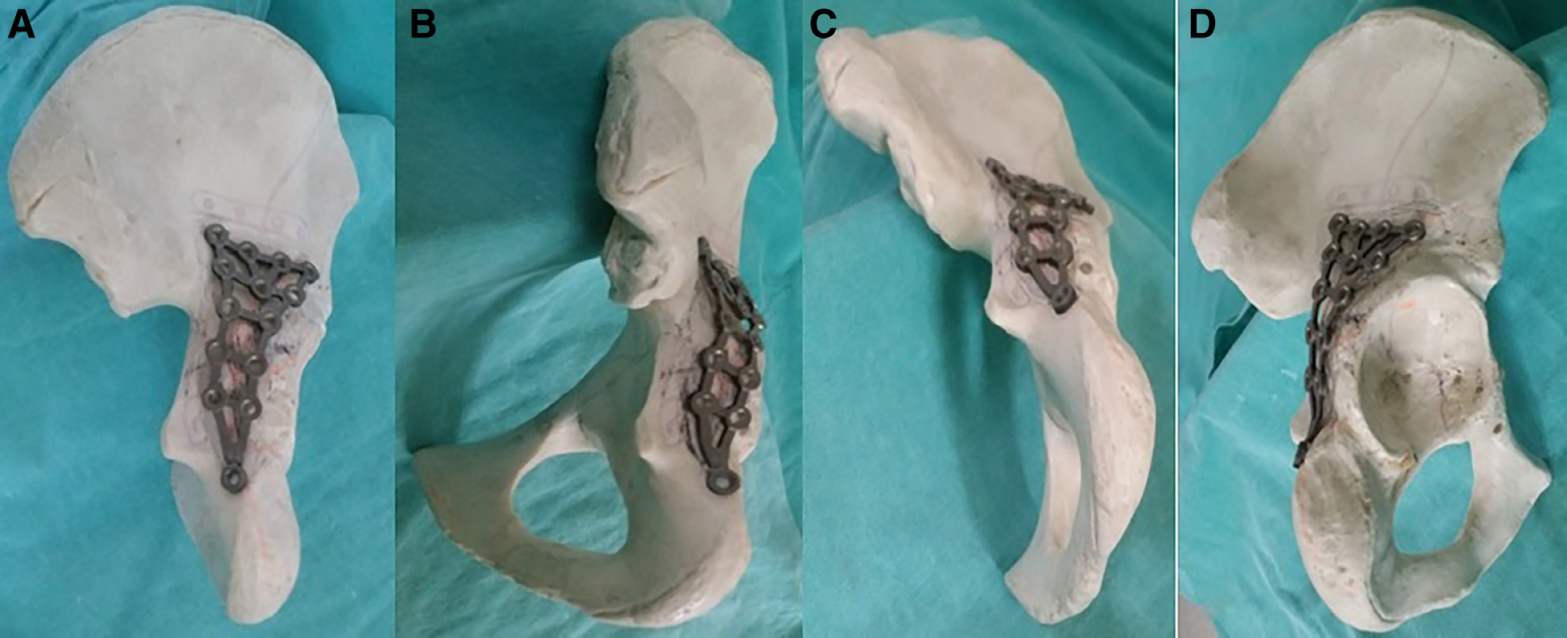
Scheme of the application of the Paseos plate to the acetabulum.

**Figure 4.**
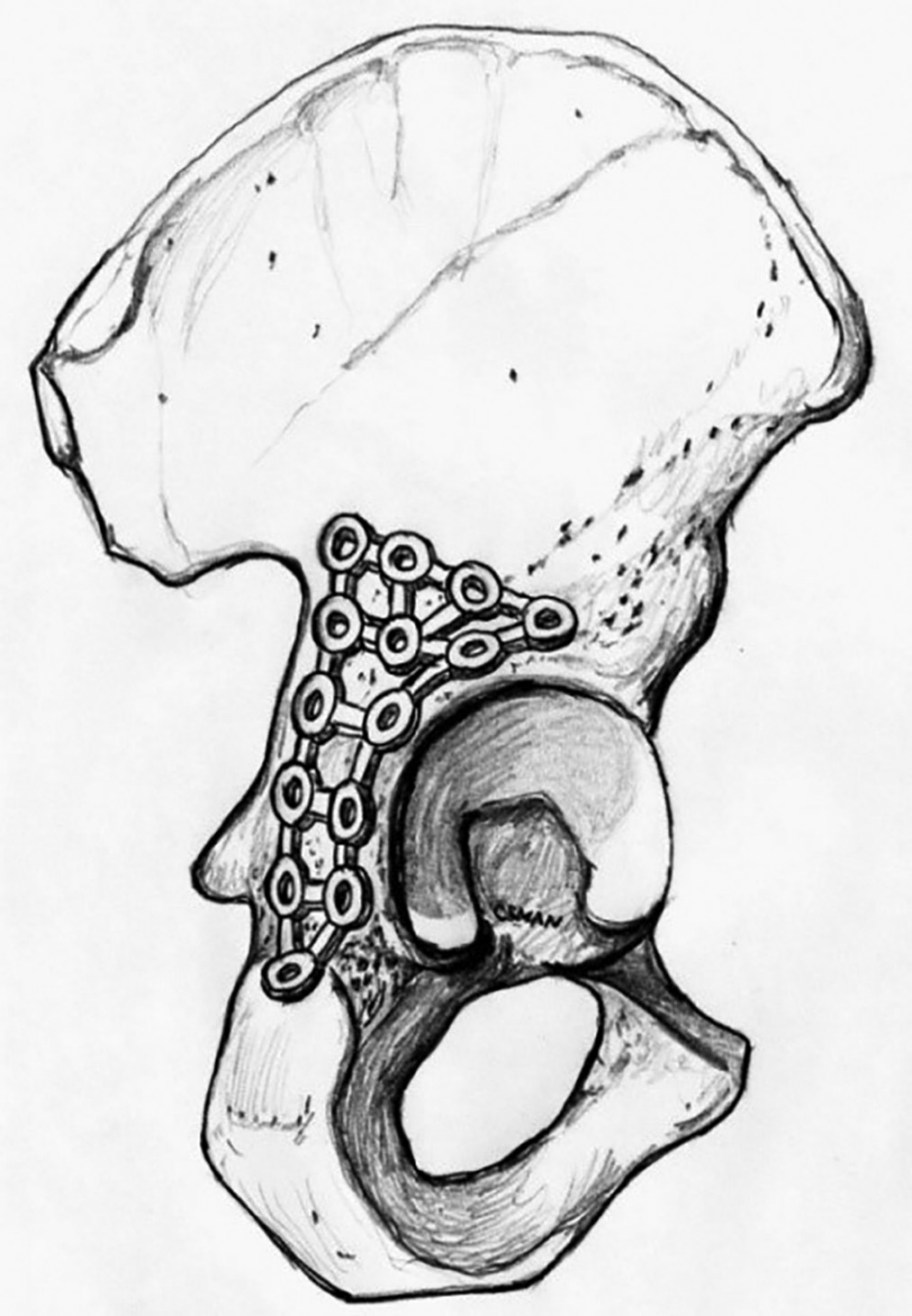
The diagram of the application of the Paseos plate on saw bone.

**Figure 5.**
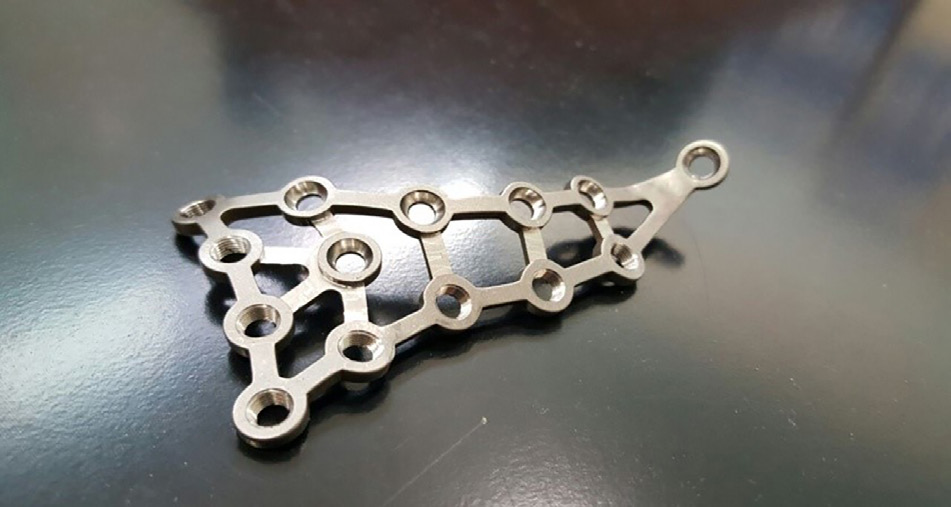
The structure of the Paseos plate.

**Figure 6.**
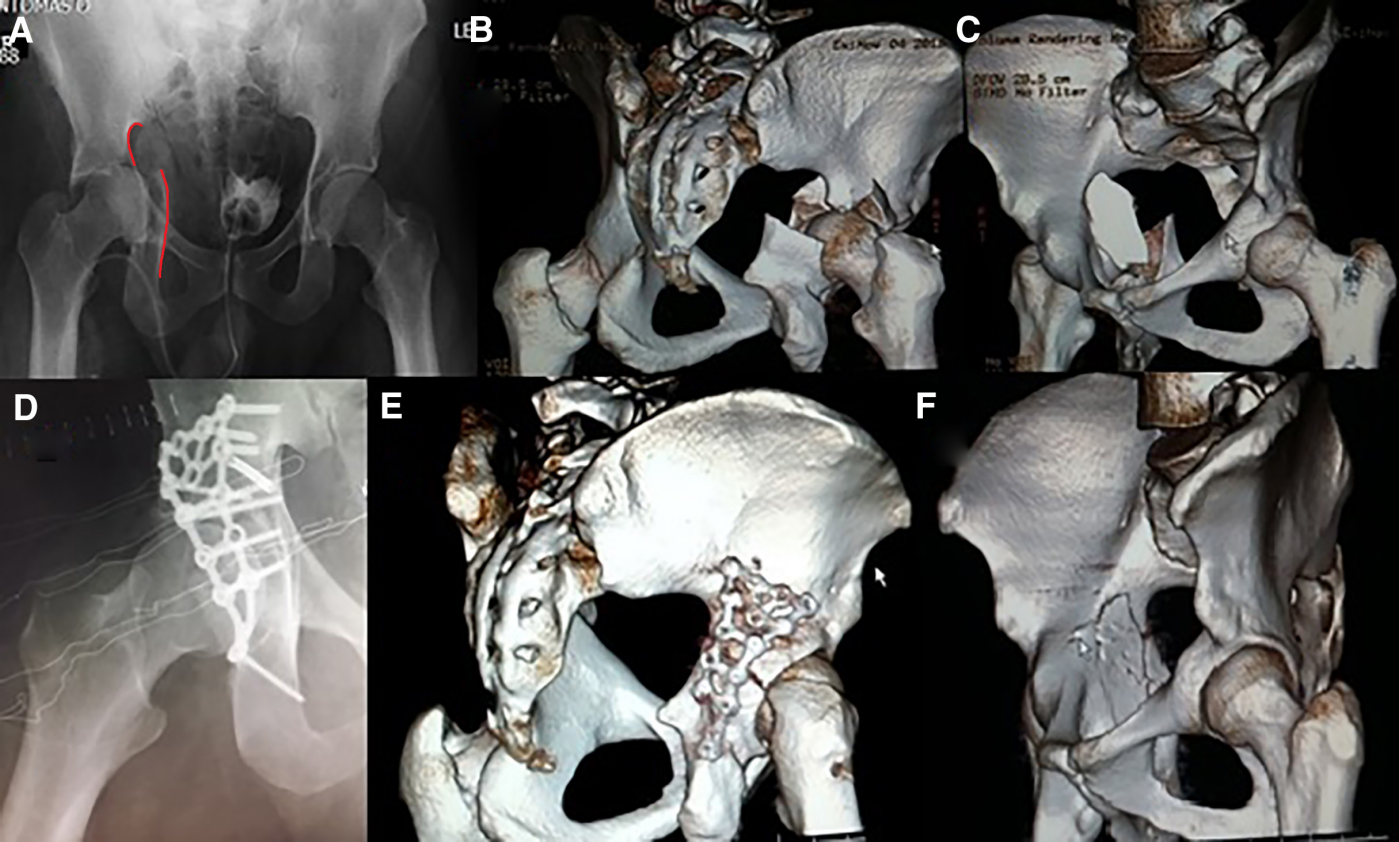
(A) Preoperative x-ray of a right posterior column and posterior wall fracture (fracture lines in red); (B, C) preoperative computed tomography images of the fracture; (D) postoperative x-ray of the Paseos plate application; (E, F) postoperative computed tomography images of the fracture.

The clinical and radiological outcomes of patients who underwent 2 different types of plate fixation for posterior acetabular wall and/or column fractures and transverse and posterior wall fractures were assessed in this study. We designed an anatomical plate that is easy to apply for acetabular posterior wall and column fractures and can simultaneously fix the column and wall as support with the buttressing effect (Figs. 3–6). During the production phase of our plate, bending and compression tests were carried out on fracture samples in the pelvis figure at Ege University Faculty of Engineering, Department of Mechanical Engineering. These preliminary biomechanical tests were performed to obtain developmental feasibility data on the plate’s load-bearing behavior. In these preliminary bending tests, the maximum force recorded before construct failure was 390 N. In the weight-bearing tests, the maximum force recorded before failure was 1800 N. The values of the applied force and stroke values of the plates are shown in Figure 2.

Demographic data, fracture types, plate types used for fixation, and postoperative MRC and MDPS are presented in Table 1. The mean age was 36.23 years in Group 1 and 34.5 years in Group 2. In Group 1, 13 patients (76.5%) were male and 4 patients (23.5%) were female. In Group 2, 12 patients (54.5%) were male and 10 patients (45.5%) were female.

**Table 1:** Comparative data and complication rates.

Variable	Group 1 (Paseos plate)	Group 2 (pelvic reconstruction plate)	*P* value*
Number of patients	17	22	–
Age (yrs), mean ± SD (median [IQR])	36.23 ± 11.85 (33 [28–37])	34.50 ± 12.70 (30.5 [25.2–36.5])	.427
Sex (Male/Female)	13/ 4	12/ 10	.193
Side of injury			1.000
- Left	9 (52.9%)	12 (54.5%)	
- Right	8 (47.1%)	10 (45.5%)	
Fracture type			.959
- Isolated posterior wall	9 (52.9%)	11 (50%)	
- Isolated posterior column	2 (11.8%)	2 (9.1%)	
- Posterior wall + column	4 (23.5%)	7 (31.8%)	
- Posterior wall + transverse	2 (11.8%)	2 (9.1%)	
Marginal impaction	5 (29.4%)	6 (27.3%)	1.000
Matta reduction criteria			1.000
- Anatomical (< 2 mm)	14 (82.3%)	19 (86.4%)	
- Good (2–3 mm)	2 (11.7%)	2 (9.1%)	
- Poor (> 3 mm)	1 (5.9%)	1 (4.5%)	
MDPS (12 mo)			.815
- Excellent	13 (76.4%)	19 (86.4%)	
- Good	3 (17.6%)	2 (9.1%)	
- Fair	1 (5.9%)	1 (4.5%)	
Mean MDPS score	17.47 ± 1.23	17.50 ± 1.33	.804
Median operation duration (IQR)	80 min (75–90)	110 min (92.5–127.5)	< .001
Complications			
- Infection	1 (5.9%)	2 (9.1%)	1.000
- Heterotopic ossification	1 (5.9%)	2 (9.1%)	1.000
- Screw loosening	0 (0%)	1 (4.6%)	1.000

* Continuous variables (age, MDPS score, operative duration) were compared using the Mann–Whitney *U* test. For categorical variables, 2 × 2 comparisons were analyzed with Fisher exact test, and comparisons involving more than 2 categories (fracture type, MRC, and MDPS categories) with the Fisher–Freeman–Halton exact test. The between-group difference in operative duration is reported as the Hodges–Lehmann median difference with its 95% CI, with the rank-biserial correlation as the effect size linked to the Mann–Whitney *U* test.

CI = confidence interval, IQR = interquartile range, MDPS: Merle d’Aubigné and Postel score, MRC = Matta reduction criteria, SD = standard deviation.

The fracture types were as follows in Group 1: isolated posterior wall (n = 9), isolated posterior column (n = 2), posterior wall and column (n = 4), posterior wall and transverse (n = 2). In Group 2, the fracture types were as follows: isolated posterior wall (n = 11), isolated posterior column (n = 2), posterior wall and column (n = 7), posterior wall and transverse (n = 2).

The reduction quality was evaluated using MRC by measuring the postoperative maximum residual displacement (MRD). The MRD was utilized to grade the quality of reduction as anatomical (MRD < 2 mm), good (MRD between 2–3 mm), and poor (MRD > 3 mm) (9). When the fracture reductions were evaluated postoperatively according to MRC; in Group 1 the reduction quality was anatomical (82.3%) in 14 patients, good in 2 patients (11.7%), and poor in 1 patient (5.9%). In Group 2, the reduction quality was anatomical (86.4%) in 19 patients, good in 2 patients (9.1%), and poor in 1 patient (4.5%). The distribution of reduction quality did not differ significantly between the 2 groups (Fisher exact test, *P* = 1.000).

The MDPS recorded in the 12-month follow-up was excellent in 13 patients (76.4%), good in 3 patients (17.6%), and fair in 1 patient (5.9%) in Group 1. The MDPS in Group 2 was excellent in 19 patients (86.4%), good in 2 patients (9.1%), and fair in 1 patient (4.5%). The mean MDPS was 17.47 ± 1.23 in Group 1 and 17.50 ± 1.33 in Group 2. There was no statistically significant difference between Group 1 and Group 2 in terms of MDPS (*P* = .804).

The median operation duration was 80 minutes (interquartile range, 75–90) in Group 1 (Paseos plate) and 110 minutes (interquartile range, 92.5–127.5) in Group 2 (reconstruction plate). The operative duration was significantly shorter in the Paseos plate group, with a Hodges–Lehmann median difference of 30 minutes (95% confidence interval, 20 to 40 minutes; Mann–Whitney U = 36.0, *P* < .001), corresponding to a large effect size (rank-biserial correlation *R* = 0.81).

Regarding complications, infection occurred in 1 patient (5.9%) in Group 1 and 2 patients (9.1%) in Group 2, heterotopic ossification in 1 (5.9%) and 2 (9.1%), and screw loosening in 0 and 1 patient (4.6%), respectively, with no significant difference between groups (all *P* = 1.000). No cases of post-traumatic arthritis, avascular necrosis of the femoral head, sciatic nerve palsy, fixation failure, reoperation, or conversion to total hip arthroplasty were observed in either group during the 12-month follow-up period.

## 4. Discussion

Achieving anatomic reduction, employing rigid internal fixation, and promoting early mobilization constitute the essential triad for successful outcomes in acetabular fractures. Experts in pelvic fracture surgery universally recognize that treatment success hinges on the precision of reduction and fixation.^[11–13]^ The other aspects of successful clinical outcomes are the configuration of the fracture, the degree of fragmentation, the energy level of the trauma, accompanying femoral head fractures, cartilage damage, and surgical experience.^[4,10,14]^

In this study, we compared the radiological and clinical results of patients operated on for posterior acetabular fractures by dividing them into 2 groups: anatomical Paseos plates and reconstruction plates. The ease and duration of the plate implementation are also evaluated. In a complex acetabular fracture series of 258 patients reported by Matta et al, anatomical reduction and fixation could be achieved in 185 (71%) of these cases. Excellent clinical results in 104 (40%) cases, good clinical results in 90 (36%) cases, unsatisfactory results in 21 (8%) cases, and poor results in 42 (16%) cases were presented. The authors emphasized that satisfactory clinical results are closely related to anatomical fracture reduction.^[9]^ In a study conducted by Gültaç et al, the clinical outcomes of 21 acetabular posterior wall fractures treated with pelvic reconstruction plates were analyzed, and the MDPS showed good and excellent results.^[15]^ In another study reported by Kilinc et al, 27 patients operated on with dual C-shaped pelvic reconstruction plates were analyzed in terms of clinical and radiological outcomes, and the authors reported 59% excellent and very good, 19% good, 11% moderate, and 11% poor results.^[3]^ When the reduction qualities and clinical outcomes provided by the pelvic reconstruction plates in the literature are examined, it is observed that radiological and clinical outcomes are parallel to our study. This relationship between reduction accuracy and outcome also underscores the importance of reliable postoperative reduction assessment. Consistent with Verbeek et al, who reported that postoperative CT assesses acetabular reduction more accurately than plain radiographs and reliably predicts hip survivorship,^[2]^ postoperative CT was obtained in all patients in the present study, strengthening the comparability of the reduction outcomes between groups.

The stability of fixation is crucial in the surgical treatment of acetabular fractures.^[13,16]^ Due to the 3D structure, fixation principles differ from those used in long bone fractures. Notably, posterior wall and column fractures, especially when accompanied by marginal impaction, require meticulous reconstruction and potential bone graft support.^[17]^ After reducing large wall fragments, the femoral head serves as a guide for joint reconstruction. While lag screw fixation is essential for large fragments, it should always be complemented by buttress plates.^[3,15,18]^ In our study, marginal impaction was detected in 5 patients in the Paseos plate group and in 6 patients in the reconstruction plate group. In all patients with marginal impaction, the joint line was reconstructed using autografts harvested from the iliac crest and fixated using the Paseos or pelvic reconstruction plates, providing a buttress effect. The 3D anatomical structure of the Paseos plate and its large proximal surface may provide, with a single implant, a bony contact area that would otherwise require several conventional reconstruction plates.

The reconstruction plates should be prepared by bending them in a way that is suitable for the patient’s bone anatomy. These plates should be placed starting from the ischiopubic ramus, and then posterior of the acetabulum and extending to the intact ilium above. In these reconstruction plates, the placement of 2 screws distally and 2 screws proximally is considered sufficient for stability.^[19,20]^ Generally, 1 or 2 pelvic reconstruction plates are used in the fixation of posterior acetabular wall or column fractures. The lateral plate is placed lateral to the acetabular posterior wall by contouring the 3.5 mm reconstruction plate into a C shape that fits the curvature of the acetabulum. Then, the second longer reconstruction plate is contoured to fit the acetabular wall and placed more medially to overlap the distal ends of the lateral plate and acetabular posterior column fixation, if necessary.^[3]^ If additional fractures extend to the iliac crest, transverse plate fixations can be applied perpendicularly to the fracture line.

In the Paseos plate, the distal end of the plate is placed on the ischial tuberosity, while the proximal part, which expands in the form of a wing, is placed towards the iliac wing, providing a buttress effect by applying pressure to the acetabular posterior wall and column region over a considerably wide area. Normally, the wide buttress effect can be achieved with multiple conventional plates; however, with this newly designed Paseos plate, a wide contact area can be achieved with just 1 single polyhedral plate. Furthermore, bending multiple conventional plates constitutes a considerable technical difficulty, which may also compromise the durability of the material. Moreover, the stress load on the fractured surfaces is distributed more evenly with a large-surface single anatomic plate. In this design, the plate includes interconnections intended to counteract both vertical shear and transverse-plane displacement; in our preliminary biomechanical tests, the construct maintained its integrity within the tested loading range. The performance of the plate in these preliminary biomechanical tests suggests that the construct can withstand loads within the tested range; however, as only 4 Paseos plate samples were tested and no reconstruction plate construct was tested for comparison, these findings represent preliminary developmental feasibility data and do not establish biomechanical superiority or clinical safety. We aimed to achieve stable fracture fixation and a buttress effect through the plate’s anatomical structure. In this way, we achieved the fixation of both the acetabular posterior wall and posterior column fractures with a single plate. In the fixation with the anatomical Paseos plate, the buttressing effect is enhanced by the plate’s anatomical design (Fig. 2). The Paseos plate is an anatomical pre-contoured plate that may reduce bending or contouring difficulties during surgery and loss of time. The median operation duration was significantly lower than the reconstruction plate group. The median operation duration was 80 minutes (75–90) in Group 1, and 110 minutes (92.5–127.5) in Group 2. Although no statistically significant differences were observed between groups regarding reduction quality or MDPS outcomes, the Paseos plate group demonstrated significantly shorter operative duration. This finding may be related to the pre-contoured anatomical structure of the implant, which reduced the need for intraoperative plate bending. The significantly shorter operative duration observed in the Paseos plate group should be interpreted cautiously. Because of the retrospective and non-randomized design, potential differences in fracture complexity, fracture morphology, and case selection between the groups may have influenced operative duration. Although similar fracture patterns were included in both groups, the possibility of selection bias cannot be completely excluded. Another important detail we noticed while using the Paseos plate is that after the screws at both ends of our plate are placed, it reduces the force applied by the femoral head from the middle of the acetabulum and, in our experience, reduced the need for separate bone clamps to hold the fragments together.

Several limitations of this study should be acknowledged. First, the retrospective and non-randomized design may have introduced selection bias and confounding related to fracture complexity, case selection, implant selection, and surgeon-related factors; although similar fracture patterns were included in both groups, these potential confounders cannot be completely excluded. All retrospective data available in the clinical records are reported; however, several potentially relevant variables (including fracture-dislocation status, associated femoral head or cartilage injury, time from injury to surgery, estimated blood loss, and fluoroscopy time) were not systematically recorded and could not be reliably reported, which further limits a full assessment of baseline comparability. In addition, the relatively small sample size and the need for longer-term follow-up represent important limitations, and because of the limited cohort size, adjusted multivariable analyses were not feasible, restricting our ability to control for possible confounding variables. Because patient allocation was time-dependent rather than randomized, a learning-curve effect cannot be excluded, and improvements in operative efficiency may partly reflect increasing surgeon experience or temporal changes in perioperative management rather than implant design alone. Furthermore, biomechanical measurements were performed only during the developmental phase of the Paseos plate, using a limited number of synthetic constructs and without a conventional reconstruction-plate comparator; therefore, no definitive conclusions regarding biomechanical superiority can be drawn. Finally, because implant selection partly depended on surgeon preference and implant availability, the findings of this study should be interpreted cautiously and considered preliminary.

## 5. Conclusion

Both fixation methods provided satisfactory clinical and radiological outcomes in posterior acetabular fractures. Although no significant differences were observed in reduction quality or MDPS outcomes, the Paseos plate was associated with shorter operative duration, possibly due to its pre-contoured anatomical design. These findings should be considered preliminary and require confirmation in larger prospective comparative studies.

## Author contributions

**Conceptualization:** Emre Gültaç, Osman Kurtuluş.

**Data curation:** Emre Gültaç, Fatih İlker Can.

**Formal analysis:** Emre Gültaç, Fatih İlker Can.

**Investigation:** Mehmet Arazi.

**Methodology:** Emre Gültaç, Fatih İlker Can, Fatih Özyer.

**Software:** Emre Gültaç, Fatih İlker Can.

**Supervision:** Emre Gültaç.

**Writing – original draft:** Emre Gültaç, Osman Kurtuluş.

**Writing – review & editing:** Mehmet Arazi.
